# Superconductivity in In-doped AgSnBiTe_3_ with possible band inversion

**DOI:** 10.1038/s41598-021-02341-9

**Published:** 2021-11-24

**Authors:** Tsubasa Mitobe, Kazuhisa Hoshi, Md. Riad Kasem, Ryosuke Kiyama, Hidetomo Usui, Aichi Yamashita, Ryuji Higashinaka, Tatsuma D. Matsuda, Yuji Aoki, Takayoshi Katase, Yosuke Goto, Yoshikazu Mizuguchi

**Affiliations:** 1 Department of Physics, Tokyo Metropolitan University, 1-1, Minami-osawa, Hachioji, 192-0397 Japan; 2 Department of Physics and Materials Science, Shimane University, 1060, Nishikawatsucho, Matsue, 690-8504 Japan; 3 Laboratory for Materials and Structures, Institute of Innovative Research, Tokyo Institute of Technology, 4259 Nagatsuta, Midori, Yokohama, 226-8503 Japan

**Keywords:** Chemistry, Materials science, Physics

## Abstract

We investigated the chemical pressure effects on structural and electronic properties of SnTe-based material using partial substitution of Sn by Ag_0.5_Bi_0.5_, which results in lattice shrinkage. For Sn_1−2*x*_(AgBi)_*x*_Te, single-phase polycrystalline samples were obtained with a wide range of *x*. On the basis of band calculations, we confirmed that the Sn_1−2*x*_(AgBi)_*x*_Te system is basically possessing band inversion and topologically preserved electronic states. To explore new superconducting phases related to the topological electronic states, we investigated the In-doping effects on structural and superconducting properties for *x* = 0.33 (AgSnBiTe_3_). For (AgSnBi)_(1−*y*)/3_In_*y*_Te, single-phase polycrystalline samples were obtained for *y* = 0–0.5 by high-pressure synthesis. Superconductivity was observed for *y* = 0.2–0.5. For *y* = 0.4, the transition temperature estimated from zero-resistivity state was 2.4 K, and the specific heat investigation confirmed the emergence of bulk superconductivity. Because the presence of band inversion was theoretically predicted, and the parameters obtained from specific heat analyses were comparable to In-doped SnTe, we expect that the (AgSnBi)_(1−*y*)/3_In_*y*_Te and other (Ag, In, Sn, Bi)Te phases are candidate systems for studying topological superconductivity.

## Introduction

Metal tellurides (*M*Te) with a NaCl-type structure have been extensively studied due to their physical properties as topological materials^1–4^, thermoelectric materials^5–7^, and superconductors^8–12^. Among them, In-doped SnTe superconductors have been drawing attention as a candidate system of a topological superconductor^4,13–17^. SnTe is a topological crystalline insulator, and superconductivity is typically induced by In-doping at the Sn site. The superconducting transition temperature (*T*_c_) of (Sn, In)Te increases by In doping. Although a simple picture proposes that doped In acts as a hole dopant, detailed analyses of carrier characteristics, superconducting properties, and electronic states of (Sn, In)Te revealed that the In doping does not simply act as a dopant of holes, but the superconductivity emerges in a regime where electron carriers are dominant^16^. Therefore, to understand the nature and the mechanisms of superconductivity in SnTe-based, development of new superconductors based on NaCl-type tellurides is important.

In *M*Te, the *M* site can be alloyed flexibly. For example, single crystals (films) of (Sn, Pb)Te can by grown with a wide solution range, and the alloy system has provided a platform to study topological nature of *M*Te^1,18,19^. Because the Pb substitution for SnTe expands the lattice, which corresponds to negative chemical pressure at the *M*–Te bond, contrasting positive chemical pressure in *M*Te leads the way for further expanding the research field of superconductivity in *M*Te. In addition, although In-doped systems of (In, Sn, Pb)Te have been studied as a topological superconductor candidate^20^, there has been no detailed study on superconducting properties and crystal structure of NaCl-type MTe with a lattice constant smaller than SnTe. In this study, we focused on the (Ag, Sn, Bi)Te system that has been studied as a thermoelectric material and found that the (Ag, Sn, Bi)Te system is possible topological material^21,22^. In SnTe, Sn is divalent, Sn^2+^. When Ag^+^_0.5_Bi^3+^_0.5_ substitutes Sn^2+^, the total valence states (charge neutrality) has been preserved. Therefore, the Ag_0.5_Bi_0.5_ substitution for the Sn site is successfully achieved in a wide range up to the end member of AgBiTe_2_. Here, we show the evolution of the structural and electronic characteristics of Sn_1−2*x*_(AgBi)_*x*_Te. Then, we report superconductivity induced by In substitution in (AgSnBi)_(1−*y*)/3_In_*y*_Te.

## Results and discussion

### Structural and electronic characteristics of Sn_1−2***x***_(AgBi)_***x***_Te

**Figure 1:**
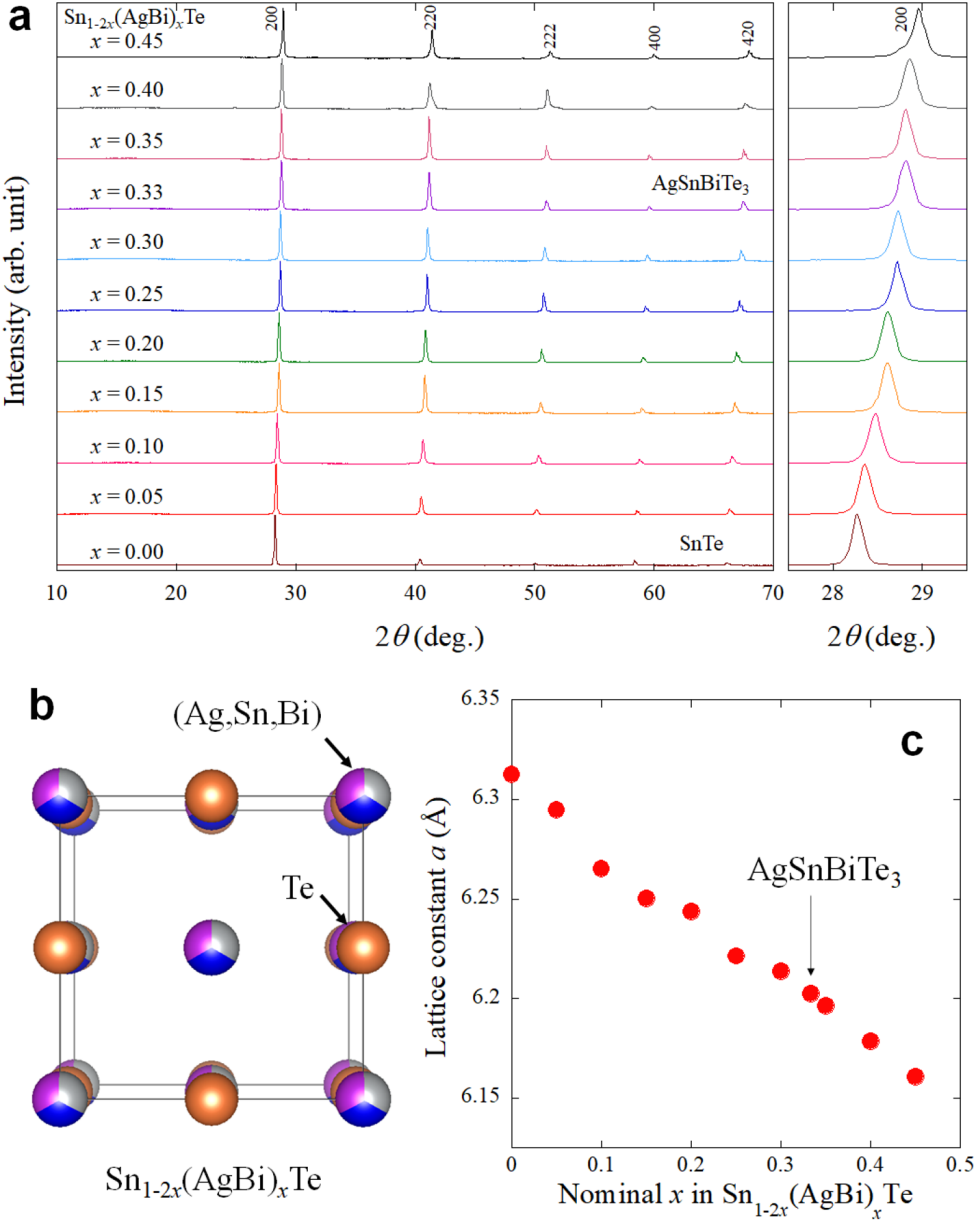
Crystal-structure evaluation of Sn_1−2*x*_(AgBi)_*x*_Te. (**a**) XRD patterns of Sn_1−2*x*_(AgBi)_*x*_Te. (**b**) Schematic image of the NaCl-type crystal structure of Sn_1−2*x*_(AgBi)_*x*_Te. (**c**) *x* dependence of the lattice constant.

As mentioned above, the (Ag, Sn, Bi)Te system would be an important system to expand the material variety of SnTe-based compounds including new superconductors. We started this work by investigating lattice compression in Sn_1−2*x*_(AgBi)_*x*_Te, in which *x* corresponds to the total amount of Ag and Bi substituted for the Sn site in the SnTe structure. Polycrystalline samples of Sn_1−2*x*_(AgBi)_*x*_Te were synthesised by a melting method. Figure 1a shows the X-ray diffraction (XRD) patterns for Sn_1−2*x*_(AgBi)_*x*_Te. The XRD peaks correspond to that expected for the NaCl-type structure (Fig. 1b) and systematically shift to higher angles, which indicates lattice shrinkage with increasing *x*. The lattice constant *a* was determined by Rietveld refinements and plotted in Fig. 1c. The trend is consistent with previous reports^20,21^. In the refinements, Ag, Sn, and Bi were assigned to the *M* site as shown in Fig. 1b, and the nominal composition (fixed) was used. Since we used laboratory XRD in this study, isotropic displacement parameter *B*_iso_ was fixed to 1 for all sites.

**Figure 2:**
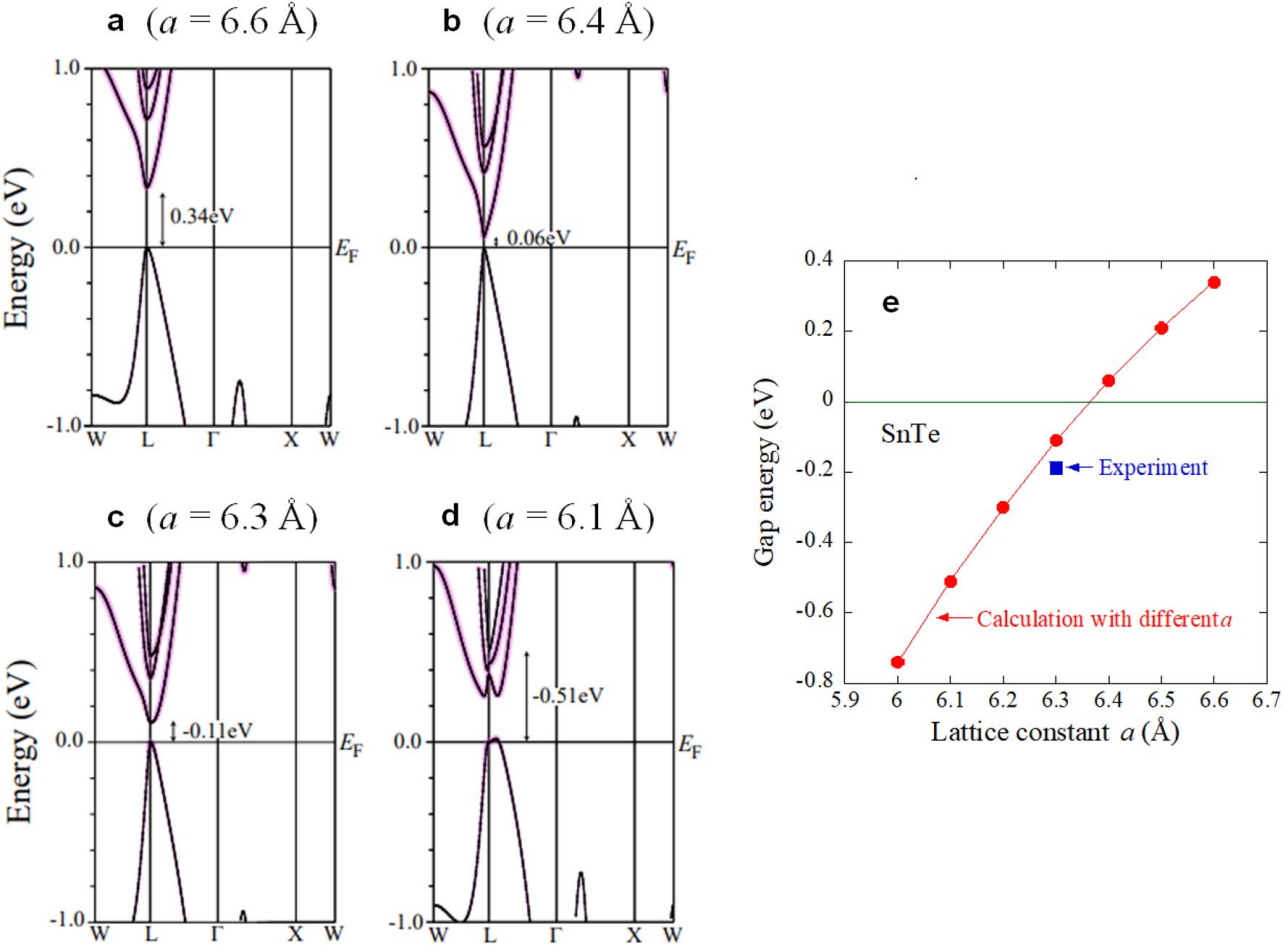
Electronic structure for SnTe with various lattice constants. (**a**–**d**) Calculated band structure. Contribution of Sn-*p* orbitals is represented by the size of pink-circle symbol. According to the calculations, a band inversion occurs with lattice constants smaller than 6.35 Å as plotted in (**e**). Experimental data in Ref.^23^ is plotted. Gap energy was calculated from the lowest energy of the Sn-p orbital and the highest energy of the Te-*p* orbital at the L point.

Figure 2 shows the calculated band structure for SnTe with various lattice constants of 6.6, 6.4, 6.3 (close to the lattice constant of SnTe), and 6.1 Å. By calculating the lowest energy of the Sn-*p* orbital and the highest energy of the Te-*p* orbital at the L point in reciprocal space, we confirmed that a band inversion transition occurs at around *a* = 6.35 Å (Fig. 2e). Note that the contributions of Sn-*p* orbitals are represented by the size of the circle symbols in Fig. 2a–d. Furthermore, calculation results with a smaller lattice constant show that SnTe-based materials with a small lattice constant of 6.1 Å also shows a band inversion. The observed trend is consistent with a previous theoretical work on SnTe and PbTe systems^2^. Therefore, we consider that the current system of Sn_1−2*x*_(AgBi)_*x*_Te basically possesses a topologically-preserved band structure with a wide range of *x*. Although calculations in this work have been performed on SnTe with different lattice constants, no obvious modification of the band structure is expected when Sn was partially replaced by Ag_0.5_Bi_0.5_ in real materials because the difference in spin–orbit interactions expected from those elements is not large. Since the orbital characteristics in the band structure does not largely change between SnTe and Sn_1−2*x*_(AgBi)_*x*_Te, we consider that the topological invariant in this system is mirror Chern number from the analogy to SnTe, which suggests that the Sn_1−2*x*_(AgBi)_*x*_Te system is a potential topological crystalline insulator. On the basis of the investigations of lattice constant and band structure for the SnTe-based system Sn_1−2*x*_(AgBi)_*x*_Te, we selected AgSnBiTe_3_ (*x* = 0.33) for a parent phase in which In-substitution effects are examined in this study.

### Superconducting properties of (AgSnBi)_(1−***y***)/3_In_***y***_Te

**Figure 3:**
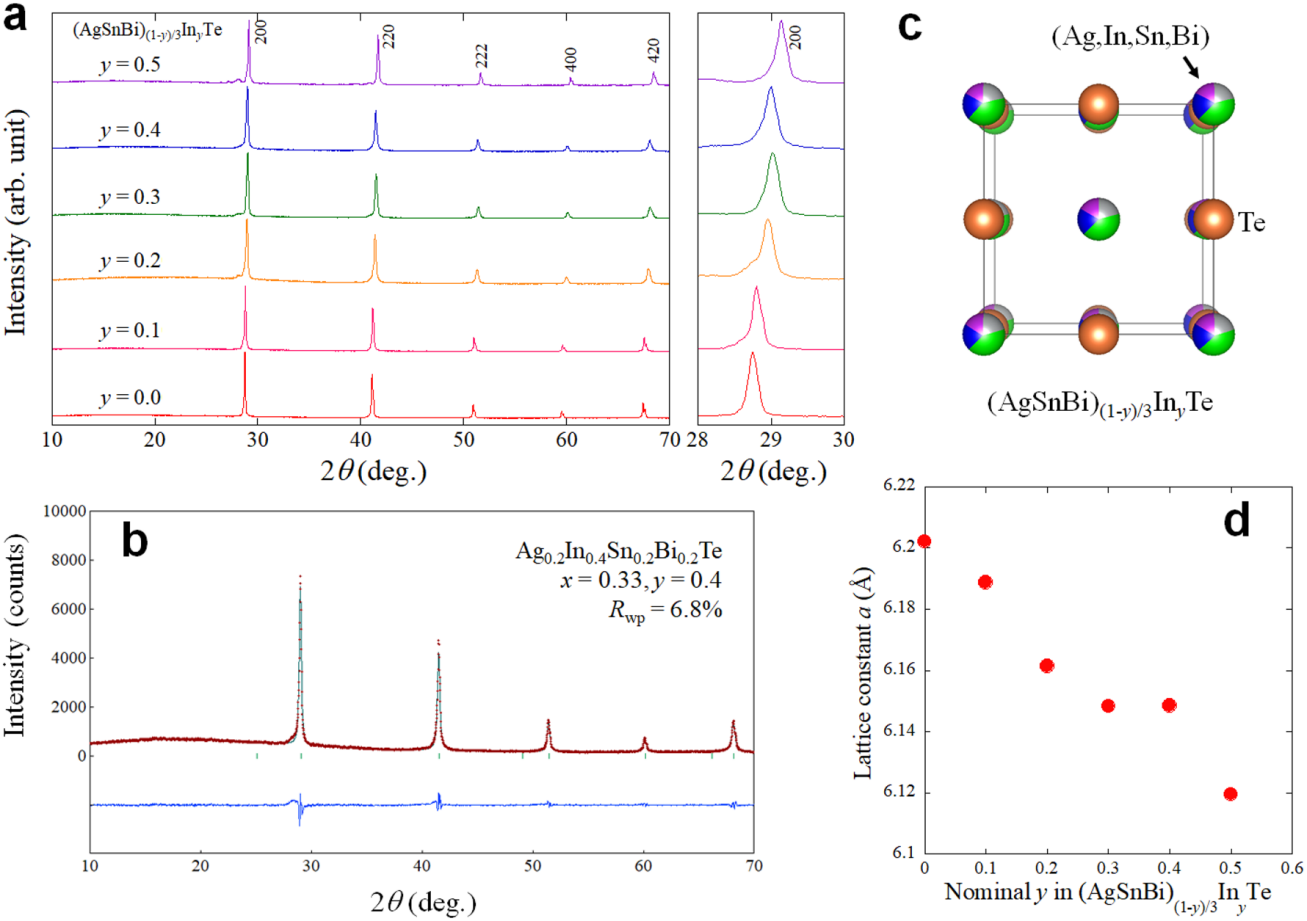
Crystal-structure evaluation of (AgSnBi)_(1−*y*)/3_In_*y*_Te. (**a**) XRD patterns of (AgSnBi)_(1−*y*)/3_In_*y*_Te. (**b**) Rietveld refinement result for *y* = 0.4. (**c**) Schematic image of the crystal structure. (**d**) *y* dependence of the lattice constant.

For the In-doped AgSnBiTe_3_ system (see Fig. 3c for crystal structure), we used a chemical formula, (AgSnBi)_(1−*y*)/3_In_*y*_Te, because the In amount doped to the parent phase of AgSnBiTe_3_ can be easily understood. Figure 3a shows the powder XRD patterns of (AgSnBi)_(1−*y*)/3_In_*y*_Te; the In-doped samples were synthesised by high-pressure annealing. As shown in Fig. 3b, Rietveld refinement reveals that tiny (shoulder) anomaly was observed. Such a shoulder structure would be due to the presence of inhomogeneous regime with a slightly different lattice constant and was observed in NaCl-type tellurides containing multiple *M*-site elements^24,25^. In particular, we tested several annealing conditions for *y* = 0.4, and the condition described in the “Methods” section was found to be the best. As shown in Fig. 3d, the lattice constant decreases with increasing *y*, which is a trend similar to other In-doped M–Te systems^8,12^. The actual element concentrations of the samples were examined by energy-dispersive X-ray spectroscopy (EDX), and the results are shown in Fig. S1 (“Supplementary information”). Basically, the actual compositions were close to the nominal values.

**Figure 4:**
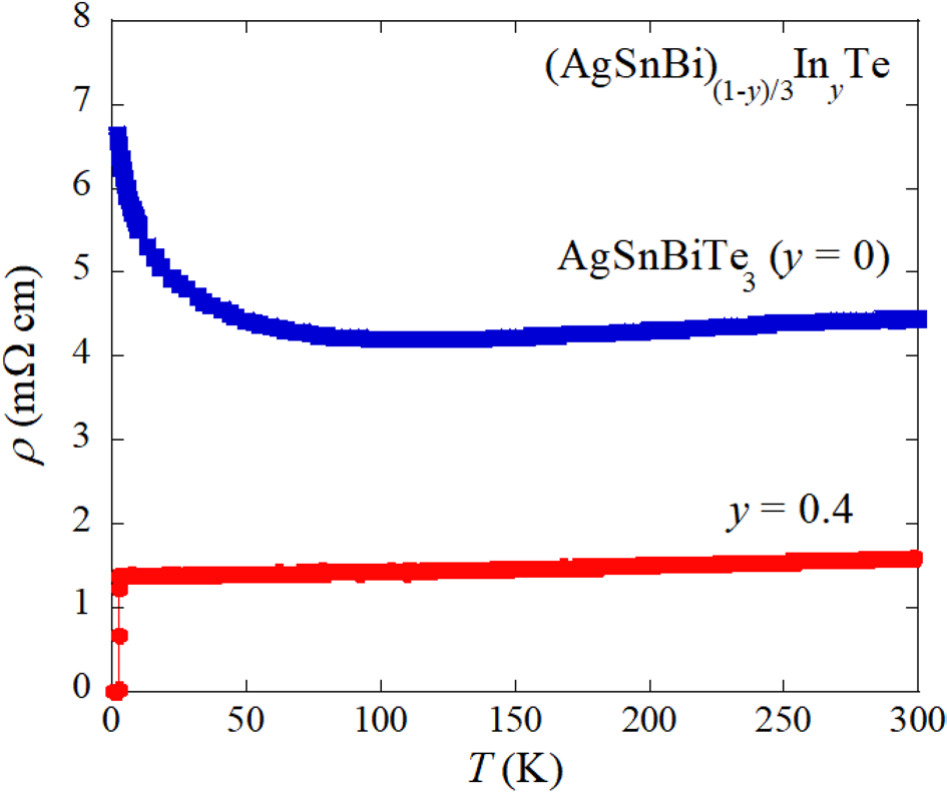
Temperature dependence of electrical resistivity for *y* = 0 (AgSnBiTe_3_) and *y* = 0.4. In-doped (*y* = 0.4) sample shows a superconducting transition at *T* = 2.4 K.

In Fig. 4, the temperature dependence of electrical resistivity of AgSnBiTe_3_ (*x* = 0.33, *y* = 0) is displayed. Resistivity slightly decreases with decreasing temperature, and an increase in resistivity was observed at low temperatures. The result is consistent with the band calculations, based on SnTe lattice, in Fig. 2, and hence, the parent phase AgSnBiTe_3_ would possess a narrow band gap. Since the calculated gap energy (Fig. 2e) indicates that a band inversion is expected for a metal telluride with *a* = 6.2 Å, AgSnBiTe_3_ with *a* = 6.20217(9) Å is expected to have topologically preserved electronic states near the Fermi energy (*E*_F_). Thus, the In-substitution effects on physical properties of AgSnBiTe_3_ are of interest because of the analogy to (Sn, In)Te, in which topological superconductivity is expected to emerge. As expected, In-doped AgSnBiTe_3_ shows superconductivity as displayed in Fig. 4. The resistivity data for *y* = 0.4 shows a metallic behavior and zero resistivity was observed at *T*_c_^zero^ = 2.4 K. For the other samples with different In concentration, the temperature dependence of resistivity are shown in Fig. S2 (“Supplementary information”). The sample with *y* = 0.1 shows almost no temperature dependence in resistivity, but other samples (*y* = 0.2–0.5) show metallic conductivity.

**Figure 5:**
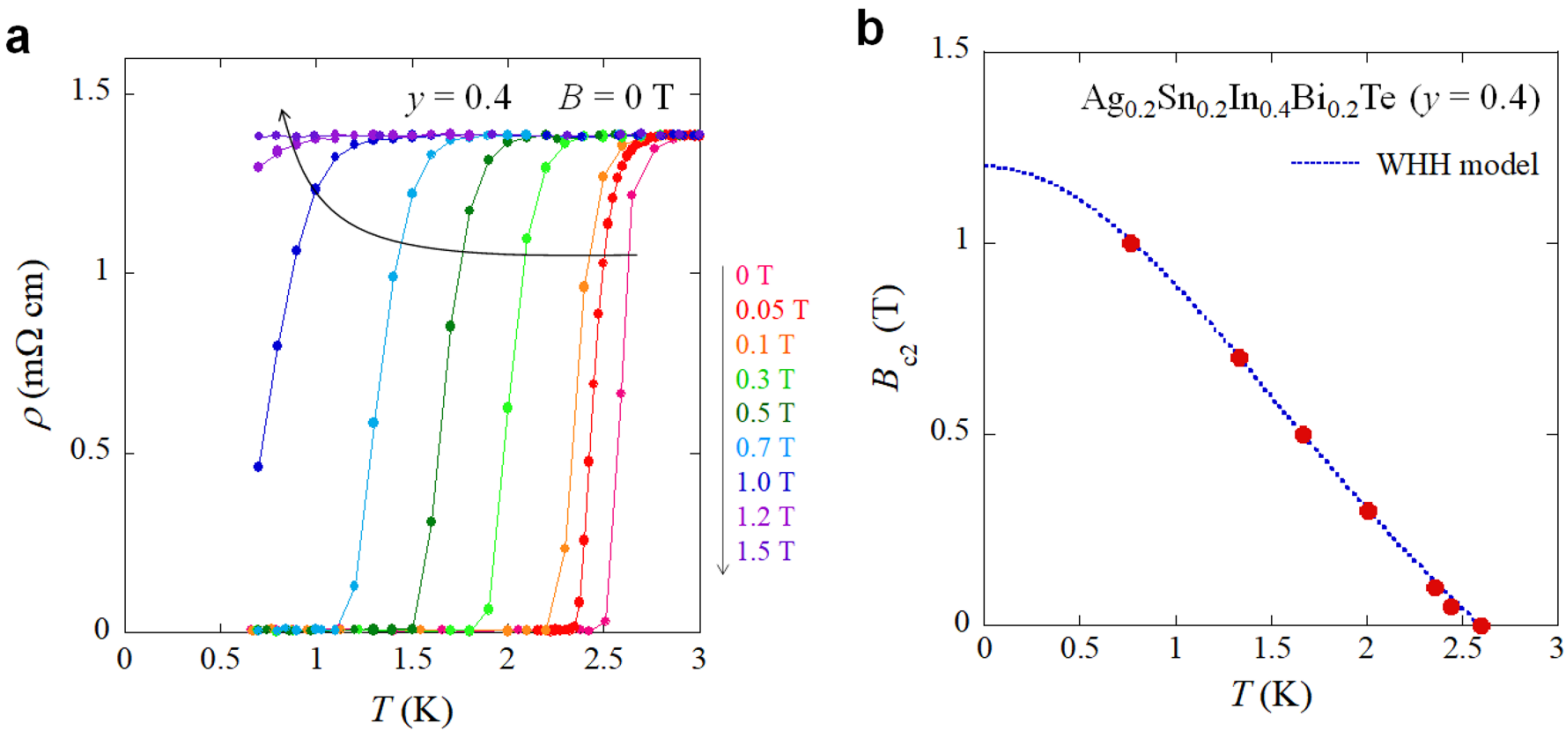
Estimation of upper critical field *B*_c2_ for *y* = 0.4. (**a**) Temperature dependences of resistivity under magnetic fields up to 1.5 T. With increasing field, the *T*_c_ systematically decreases. (**b**) Temperature dependence of *B*_c2_. For the plot of *B*_c2_, middle-point temperatures, where resistivity became 50% of normal-state resistivity at 3 K, were estimated in (**a**). The dotted line shows WHH fitting.

To investigate upper critical field (*B*_c2_), temperature dependences of resistivity were measured under various magnetic fields up to 1.5 T as plotted in Fig. 5a. For the estimation of *B*_c2_, the transition temperature was determined as a temperature where resistivity drops to 50% of the normal-state resistivity. Using the WHH model (Werthamer–Helfand–Hohenberg model)^26^, which is applicable for a dirty-limit type-II superconductor, the *B*_c2_(0) was estimated as 1.2 T, as shown in Fig. 5b.

**Figure 6:**
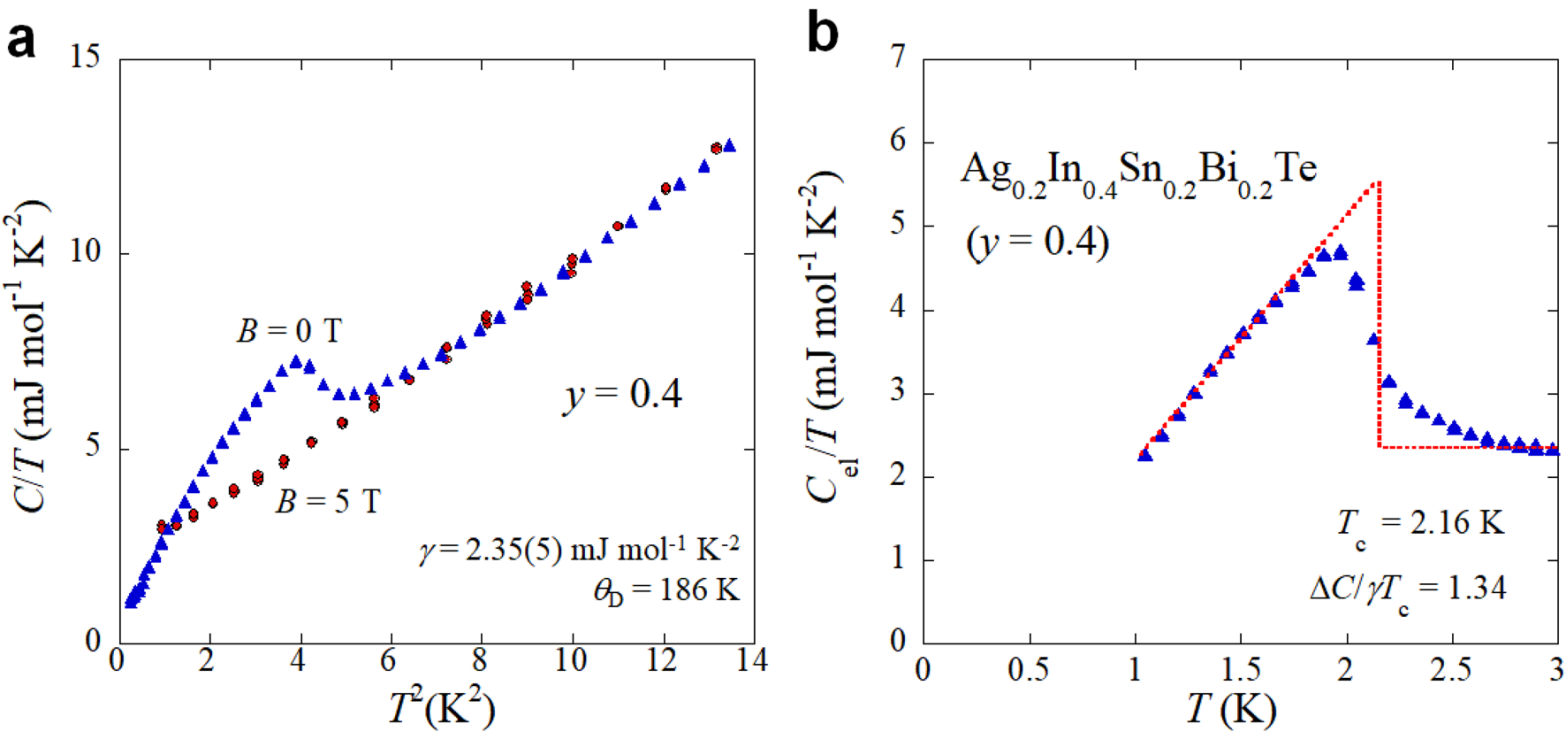
Specific heat data for *y* = 0.4. (**a**) Specific heat (*C*/*T*) data under magnetic fields (*B*) of 0 and 5 T plotted as a function of *T*^2^. By fitting the data under *B* = 5 T, electronic specific heat parameter (*γ*) and Debye temperature (*θ*_D_) were estimated. (**b**) Temperature dependence of the electronic contribution (*C*_el_/*T*) under *B* = 0 T. The electronic contribution was calculated by subtracting the lattice contribution. *T*_c_ and the superconducting jump at *T*_c_ (Δ*C*) were estimated by considering the entropy balance, as guided by the red dashed lines. Δ*C*/*γT*_c_ = 1.34, which is close to BCS value of 1.43.

Figure 6 shows the analysis results of specific heat measurements. In Fig. 6a, specific heat data under 0 and 5 T in a form of *C*/*T* are plotted as a function of *T*^2^. From the data under 5 T, the electronic specific heat parameter (*γ*) and the Debye temperature (*θ*_D_) were estimated as 2.35(5) mJ mol^−1^ K^−2^ and 186 K, respectively. The low-temperature specific-heat formula with anharmonic term was used for the analysis: *C* = *γT* + *βT*^3^ + *δT*^5^, where *βT*^3^ is the lattice contribution to the specific heat, and the *δT*^5^ term accounts for anharmonicity of the lattice. The *θ*_D_ was calculated from *β* = (12/5)π^4^(2*N*)*k*_B_*θ*_D_^−3^, where *N* and *k*_B_ are the Avogadro constant and Boltzmann constant, respectively. To characterize the superconducting properties, the electronic contribution (*C*_el_) under 0 T, which was calculated by subtracting lattice contributions from the specific heat data under 0 T_,_ is plotted in the form of *C*_el_/*T* as a function of temperature in Fig. 6b. The clear jump of *C*_el_/*T* and decrease in *C*_el_/*T* at low temperatures suggest the emergence of bulk superconductivity. The superconducting jump in *C*_el_ (Δ*C*_el_) estimated with *T*_c_ = 2.16 K is 1.34*γT*_c_, which is comparable to the value expected from a full-gap superconductivity based on the BCS model (Δ*C*_el_ = 1.43*γT*_c_)^27^. Those values obtained from specific heat are similar to those obtained for In-doped SnTe and Ag-doped SnTe superconductors with 20% In (or Ag) doping^8,28^.

**Figure 7:**
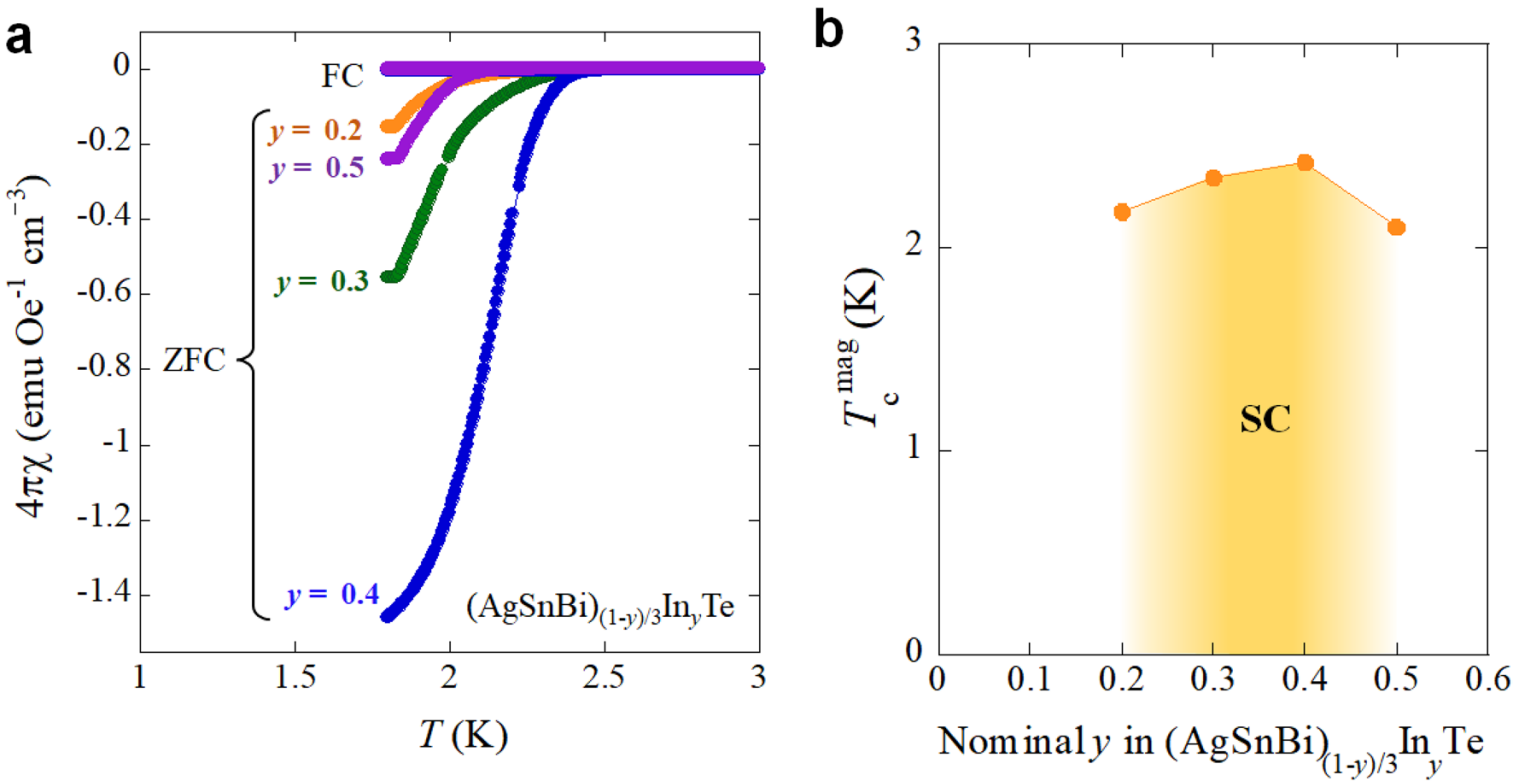
Magnetic susceptibility and superconducting transition temperature for (AgSnBi)_(1−*y*)/3_In_*y*_Te. (**a**) Temperature dependences of magnetic susceptibility (4π*χ*) for *y* = 0.2, 0.3, 0.4, 0.5. (**b**) In concentration (*y*) dependence of *T*_c_ estimated from 1% shielding volume fraction (*T*_c_^mag^).

To investigate the In-doping dependence of superconducting states, temperature dependences of magnetic susceptibility (4π*χ*) was measured for (AgSnBi)_(1−*y*)/3_In_*y*_Te. For, *y* = 0 and 0.1, no superconducting transition was observed at temperatures above 1.8 K. Superconducting diamagnetic signals were observed for *y* = 0.2–0.5, as displayed in Fig. 7a. Particularly, samples with *y* = 0.3 and 0.4 showed a large shielding fraction. Note that the data has not been corrected by demagnetisation effect. The observation of the largest shielding volume fraction is consistent with the bulk superconductivity confirmed in specific heat measurements. As shown in Fig. 7b, *T*_c_ estimated from magnetic susceptibility (*T*_c_^mag^) becomes the highest at *y* = 0.4 and decreases with further In doping (*y* = 0.5).

**Figure 8:**
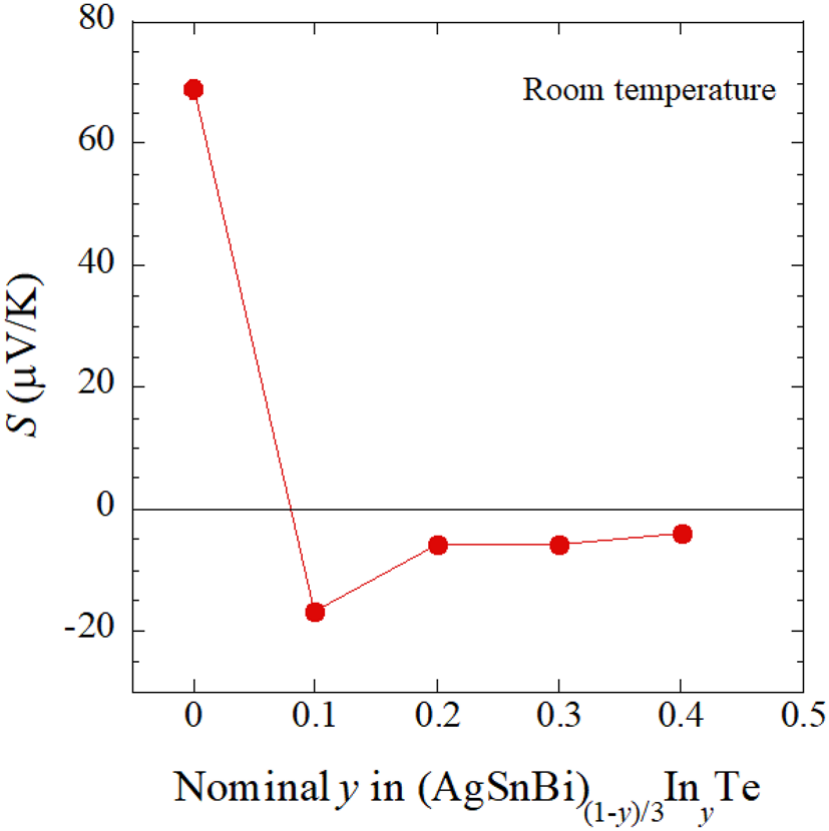
Effect of In doping on Seebeck coefficient (*S*) for (AgSnBi)_(1−*y*)/3_In_*y*_Te. In concentration (*y*) dependence of S at room temperature is shown.

The estimated *T*_c_ for *y* = 0.4 is lower than that for (Sn, In)Te (*T*_c_ ~ 4.5 K)^16^. The difference may be caused by three possible reasons: (1) carrier concentration, (2) the effect of disorder, and (3) lattice constant. On (1) carrier concentration, we performed measurements of Seebeck coefficient (at room temperature) and Hall coefficient (at 5 K for *y* = 0.4). Figure 8 shows the *y* dependence of Seebeck coefficient (*S*). For *y* = 0, a large positive value of *S* was observed. This is consistent with the band calculation, indicating that the parent phase of *y* = 0 is a semiconductor. With increasing *y*, *S* becomes negative, and the absolute value for *y* > 0.1 becomes less than 10 μV K^−1^, which is a typical value of metals. In Fig. S3 (“Supplementary information”), the magnetic field dependence of Hall resistance at 5 K is plotted. By linear fitting of the data and assuming a single-band model, carrier concentration was calculated as 7.4 × 10^21^ cm^−3^. These results on the evolution of carrier concentration by In substitution in (AgSnBi)_(1−*y*)/3_In_*y*_Te would suggest that electrons are doped by In^3+^ substitution, and the doping situation in the present system is clearly different from that observed in (Sn, In)Te^16^. Therefore, to investigate the relationship between superconducting properties and carrier concentration in (AgSnBi)_(1−*y*)/3_In_*y*_Te, further investigation with various probes is needed. On the effect of (2) disorder, it is a fact that high configurational entropy of mixing is present at the *M* site as described in the following discussion. However, comparison of *T*_c_ between *y* = 0.4 (*M* = Ag, In, Sn, Bi) and other *M*Te superconductors, for example, *M* = In (*T*_c_ ~ 3 K)^9,12^ and *M* = Sn_0.8_Ag_0.2_ (*T*_c_ = 2.3 K)^11^, suggests that the higher disorder in *y* = 0.4 (*M* = Ag, In, Sn, Bi) is not highly affecting *T*_c_. On the effect of (3) lattice constant, the electronic structure of SnTe-based materials is modified by lattice constant as shown above. In addition, in Ref.^25^, we showed that *T*_c_ of *M*Te shows a positive relation to lattice constant. Therefore, the *T*_c_ obtained for *y* = 0.4 would be reasonable to the trend of *T*_c_-lattice constant for *M*Te. According to those facts, we consider that the difference in *T*_c_ between *y* = 0.4 and (Sn, In)Te is caused by the difference in the electronic states (carrier concentration and/or lattice constant).

### Solubility limit and phase stabilisation by configurational entropy of mixing

The doping phase diagram for superconductivity of (AgSnBi)_(1−*y*)/3_In_*y*_Te is compared with that for the (Sn, In)Te and (Pb, In)Te systems in the following discussion. Having looked at the phase diagrams of (Sn, In)Te and (Pb, In)Te^8,12^, superconductivity is observed in a wide range of In concentration. As shown in Fig. 7b, however, the superconducting properties (*T*_c_ and shielding volume fraction) becomes the highest for *y* = 0.4, and superconductivity seems to be suppressed for further In doping. Therefore, the trend of In-doping effect on superconductivity in the AgSnBiTe_3_ system is different from that in the SnTe and PbTe systems. We consider that the suppression of superconductivity is caused by the solution limit of In rather than the changes in electronic states.

**Figure 9:**
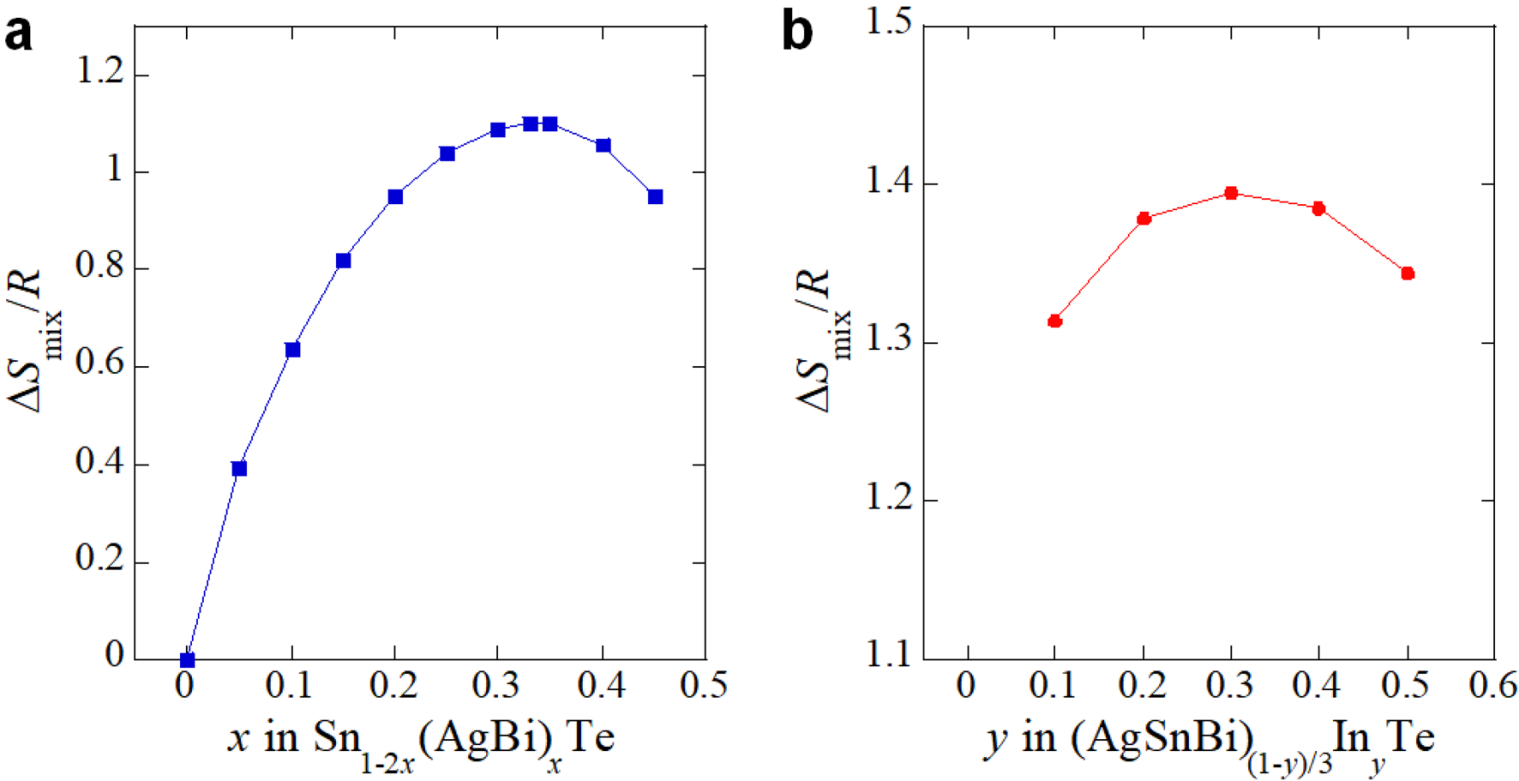
Configurational entropy of mixing (Δ*S*_mix_). (**a**) *x* dependence of Δ*S*_mix_ for Sn_1−2*x*_(AgBi)_*x*_Te. (**b**) *y* dependence of Δ*S*_mix_ for (AgSnBi)_(1−*y*)/3_In_*y*_Te.

We tried to synthesise samples with *y* > 0.5, but a high-purity sample was not obtained. In addition, the Bi amount for *y* = 0.5 deviates from the nominal value with a large error, which means that the sample with *y* = 0.5 also contains inhomogeneity larger than those in *y* < 0.5. Seeing lattice constants for In-doped samples, we find that the lattice constant is smaller than the end member of InTe with *a* ~ 6.175 Å^8^. Therefore, we consider that the suppression of superconductivity observed for *y* = 0.5 is due to the increase in inhomogeneity in the sample, and the solution limit of In for AgSnBiTe_3_ is around *y* = 0.4 under pressures up to 2 GPa. As a fact, *y* = 0.4 samples show degradation of superconducting properties by passing time. Thus, in this study, investigations of superconducting properties of *y* = 0.4 have been performed in 24 h after the high-pressure synthesis. Because the (Ag,In,Sn,Bi)Te system is a kind of disordered system with high configurational entropy of mixing (Δ*S*_mix_), we briefly discuss the possible explanation of the solution limit of In in (Ag, In, Sn, Bi)Te. As established in a field of high-entropy alloys, the phase of multiple-element system can be stabilized owing to high Δ*S*_mix_^29,30^, which decreases Gibbs free energy, Δ*G* = Δ*H* − *T*Δ*S*, at high temperatures, where *H* is enthalpy. Δ*S*_mix_ values for Sn_1−2*x*_(AgBi)_*x*_Te and (AgSnBi)_(1−*y*)/3_In_*y*_Te were calculated using Δ*S*_mix_ = − *R*Σ^*i*^*c*_*i*_ln*c*_*i*_, where *c*_*i*_ and *R* are the atomic fraction of component *i* and the gas constant, respectively. As shown in Fig. 9a, for Sn_1−2*x*_(AgBi)_*x*_Te, Δ*S*_mix_ is relatively high with a wide range of *x*, which would be the reason why the phases could be obtained with a wide range of *x* without the use of high-pressure synthesis. For In doping, we selected AgSnBiTe_3_ (*x* = 0.33) as a parent phase in this study. For (AgSnBi)_(1−*y*)/3_In_*y*_Te, Δ*S*_mix_ becomes the highest at *y* = 0.3, and it decreases with further In substitution, as shown in Fig. 9b. Therefore, the possible explanation of the solution limit of In for AgSnBiTe_3_ is as follows. Due to the small lattice constant of ~ 6.2 Å for AgSnBiTe_3_, In substitution was challenging, but the phase was stabilized up to *y* = 0.4 by the use of high pressure and high Δ*S*_mix_. If we could get a phase with a higher In concentration, the superconducting phase diagram would be expanded from that shown in Fig. 7b.

## Summary

We have synthesised polycrystalline samples of Sn_1−2*x*_(AgBi)_*x*_Te and (AgSnBi)_(1−*y*)/3_In_*y*_Te to explore a new candidate phase of topological superconductor. For Sn_1−2*x*_(AgBi)_*x*_Te, single-phase samples were obtained with a wide range of *x*. According to band calculations, we confirmed that the Sn_1−2*x*_(AgBi)_*x*_Te system is basically possessing band inversion and topologically preserved electronic states. To investigate the effects of In substitution, we selected *x* = 0.33 (AgSnBiTe_3_) as a parent phase. For (AgSnBi)_(1−*y*)/3_In_*y*_Te, In-doped samples were obtained for *y* = 0–0.5 by high-pressure synthesis, and superconductivity was observed for *y* = 0.2–0.5. For *y* = 0.4, specific heat investigation confirmed the emergence of bulk superconductivity. Although the current study is the material exploration with polycrystalline samples, we expect that the single crystals of (Ag,In,Sn,Bi)Te are grown in the next step, and characteristics including surface states, which are expected for a topological superconductor, are experimentally examined by surface-sensitive probes like angle-resolved photoemission spectroscopy.

## Methods

Polycrystalline samples of Sn_1−2*x*_(AgBi)_*x*_Te (*x* = 0.00, 0.05, 0.10, 0.15, 0.20, 0.25, 0.30, 0.33, 0.35, 0.40, 0.45) and (AgSnBi)_(1−*y*)/3_In_*y*_Te (*y* = 0.0, 0.1, 0.2, 0.3, 0.4, 0.5) were prepared by a melting method in an evacuated quartz tube. Powders or grains of Ag (99.9% up), Sn (99.99%), Bi (99.999%), In (99.99%), and Te (99.999%) were mixed and melted in an evacuated quartz tube at 900 °C for 10 h, followed by furnace cooling to room temperature. For (AgSnBi)_(1−*y*)/3_In_*y*_Te, the obtained samples were powdered and annealed in a high-pressure-synthesis instrument under 2 GPa at 500 °C for 30 min. A cubic-anvil-type 180-ton press was used, and the sample sealed in a BN crucible was heated by carbon heater.

The phase purity and the crystal structure of Sn_1−2*x*_(AgBi)_*x*_Te and (AgSnBi)_(1−*y*)/3_In_*y*_Te were examined by laboratory X-ray diffraction (XRD) by the *θ*–2*θ* method with a Cu–K_α_ radiation on a MiniFlex600 (RIGAKU) diffractometer equipped with a high-resolution detector D/tex Ultra. The schematic images of crystal structures were drawn by VESTA^31^ using a structural data refined by Rietveld refinement using RIETAN-FP^32^. The actual compositions of the examined samples were analysed using an energy dispersive X-ray spectroscopy (EDX) on TM-3030 (Hitachi).

The temperature dependence of magnetic susceptibility was measured using a superconducting quantum interference devise (SQUID) on MPMS-3 (Quantum Design) after zero-field cooling (ZFC) with an applied field of 10 Oe. The temperature dependence of electrical resistivity was measured by a four-probe method with an applied DC current of 1 mA on PPMS (Quantum Design) under magnetic fields. We used Ag paste and Au wires (25 μm in diameter) for the four-probe setup. The temperature dependence of specific heat was measured under 0 and 5 T by a relaxation method on PPMS. The resistivity and specific heat measurements were performed using a ^3^He probe system (Quantum Design). Hall coefficient was measured by four-probe setup on PPMS at low temperatures. Hall coefficient was estimated from the slope in the magnetic field dependence of Hall voltage. Seebeck coefficient at room temperature was measured under steady-state, where the thermo-electromotiveforce (∆*V*) and the temperature difference (∆*T*) were simultaneously measured, and the *S* was determined from the slope of ∆V/∆*T*.

First principles band calculations were performed using WIEN2k package^33^. The electronic density of PbTe and SnTe was self-consistently calculated within the modified Becke–Johnson potential^34^ using a 12 × 12 × 12 k-mesh and *RK*_max_ = 9 with the spin–orbit coupling included.

## Supplementary Information

Supplementary Figures.
