# Supplementary material for: Superconductivity in In-doped AgSnBiTe3 with possible band inversion
Source: Sci Rep. 2021 Nov 24;11:22885. doi: 10.1038/s41598-021-02341-9 (PMC8613227; doi:10.1038/s41598-021-02341-9)
Supplement: Supplementary file 1 — Supplementary Figures. [file 41598_2021_2341_MOESM1_ESM.pdf]

Supplementary information

Tsubasa Mitobe[1], Kazuhisa Hoshi[1], Md. Riad Kasem[1], Ryosuke Kiyama[1], Hidetomo Usui[2], Aichi Yamashita[1], Ryuji Higashinaka[1], Tatsuma D. Matsuda[1], Yuji Aoki[1], Takayoshi Katase[3], Yosuke Goto[1], Yoshikazu Mizuguchi[1]*

[1]Department of Physics, Tokyo Metropolitan University, 1-1, Minami-osawa, Hachioji 192-0397

[2]*Department of Physics and Materials Science, Shimane University, 1060, Nishikawatsucho, Matsue 690-8504, Japan.*

[3]*Laboratory for Materials and Structures, Institute of Innovative Research, Tokyo Institute of Technology, Nagatsuta, Midori, Yokohama 226-8503, Japan*

Corresponding author: Yoshikazu Mizuguchi (mizugu@tmu.ac.jp)

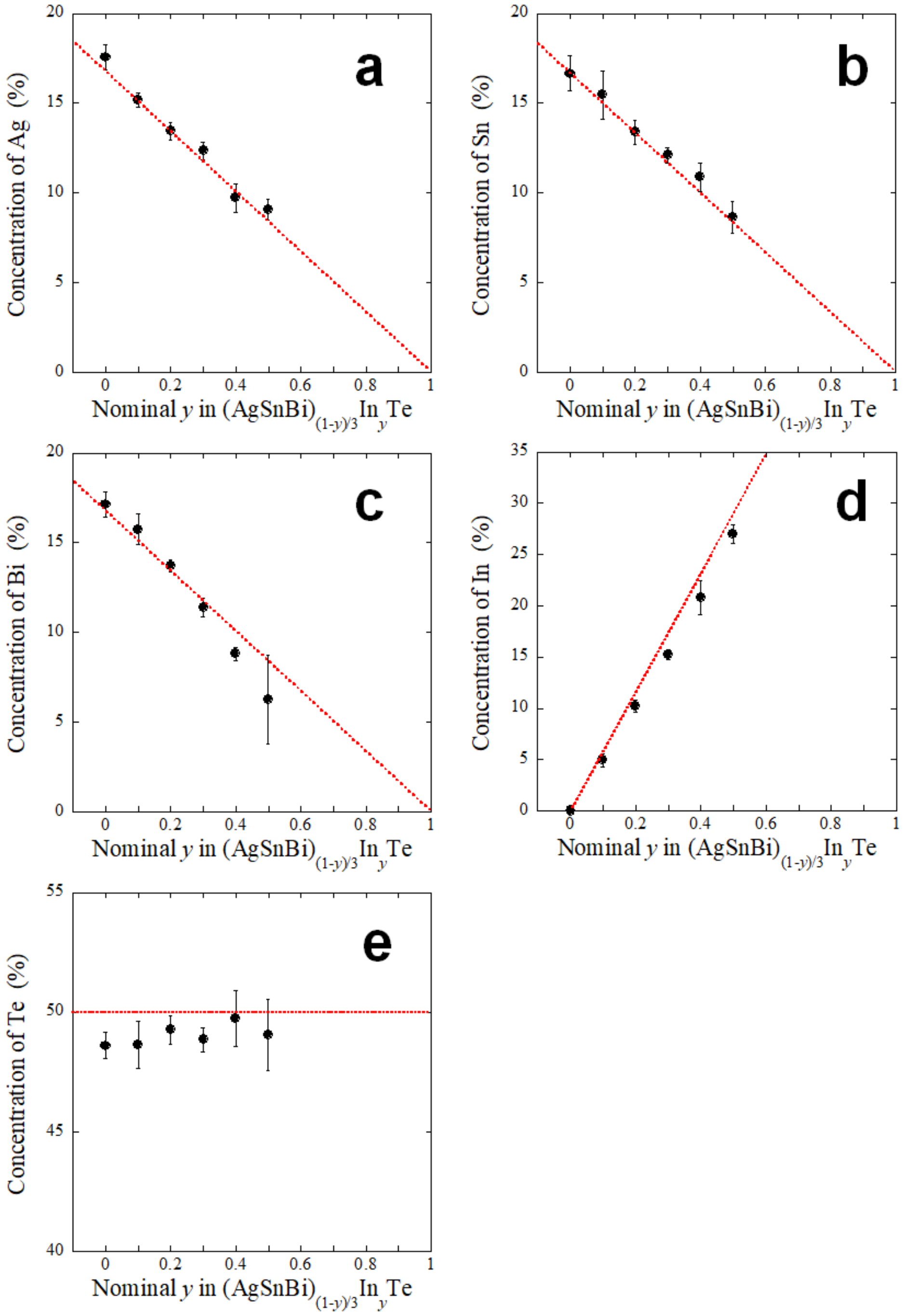

Fig. S1. EDX analysis results for $(AgSnBi)_{(1-y)/3}In_yTe$. Red dot lines indicate an ideal composition.

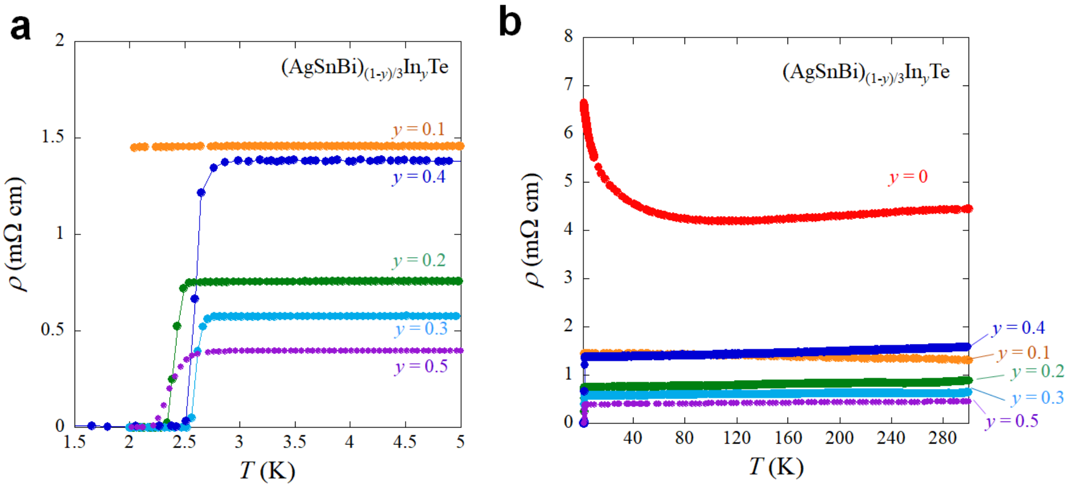

Fig. S2. Temperature dependences of electrical resistivity (ρ) for $(AgSnBi)_{(1-y)/3}In_yTe$.

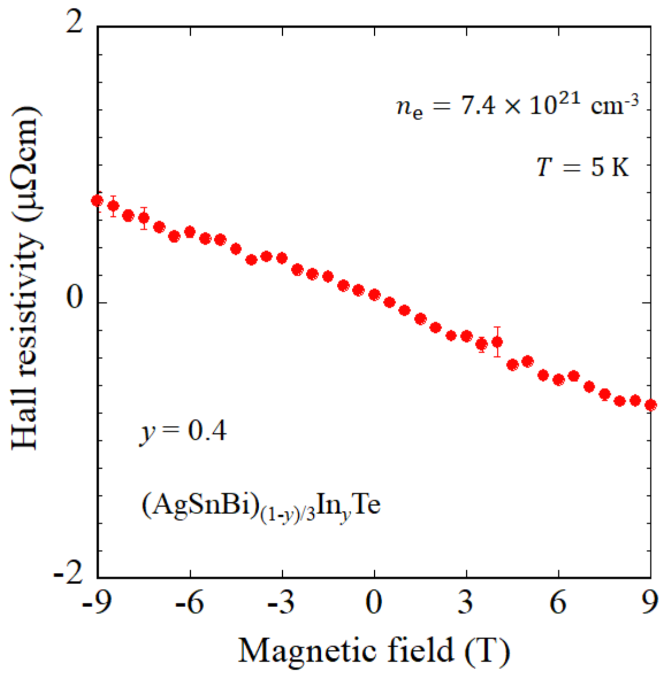

Fig. S3. Magnetic field dependences of Hall resistivity at 5 K for $y = 0.4$.
